# Incidence and Outcomes Associated With *Clostridioides difficile* Infection in Solid Organ Transplant Recipients

**DOI:** 10.1001/jamanetworkopen.2021.41089

**Published:** 2021-12-29

**Authors:** Seyed M. Hosseini-Moghaddam, Bin Luo, Sarah E. Bota, Shahid Husain, Michael S. Silverman, Nick Daneman, Kevin A. Brown, J. Michael Paterson

**Affiliations:** 1 ICES, Ontario, Canada; 2 Multiorgan Transplant Program, University Health Network, University of Toronto, Toronto, Ontario, Canada; 3 Division of Infectious Diseases, Department of Medicine, Western University, London, Ontario, Canada; 4 Sunnybrook Health Sciences Center, University of Toronto, Toronto, Ontario, Canada; 5 Institute of Health Policy, Management, and Evaluation, University of Toronto, Toronto, Ontario, Canada; 6 Public Health Ontario, Toronto, Ontario, Canada; 7 Dalla Lana School of Public Health, Toronto, Ontario, Canada; 8 Department of Family Medicine, McMaster University, Hamilton, Ontario, Canada

## Abstract

**Importance:**

Little is known about the incidence and outcomes of *Clostridioides difficile* infection (CDI) in solid organ transplant (SOT) recipients.

**Objective:**

To estimate the CDI incidence and outcomes in SOT recipients.

**Design, Setting, and Participants:**

A population-based cohort study was conducted using administrative health care data for all Ontario, Canada, residents who received organ allografts from April 1, 2003, to December 31, 2017; March 31, 2020, was the end of the study period.

**Main Outcomes and Measures:**

The primary outcome was hospital admission with CDI diagnosis. The secondary outcomes included all-cause death, intensive care unit admission, acute kidney injury requiring dialysis, and fulminant CDI comprising any of the following: toxic megacolon, ileus, perforation, or colectomy. The association between short- vs long-term mortality (ie, death occurring within or after 90 days post-CDI) and the following variables was evaluated: age, sex, Deyo-Charlson Comorbidity Index, SOT type, early- vs late-onset CDI, fulminant CDI, intensive care unit admission, and acute kidney injury requiring acute dialysis.

**Results:**

Overall, 10 724 SOT recipients (6901 [64.4%] men; median age, 54 [IQR, 44-62] years) were eligible. Kidney transplant was the most common SOT type (6453 [60.2%]). The median follow-up time was 5.0 (IQR, 2.3-8.8) years, resulting in 61 987 person-years of follow-up. A total of 726 patients (6.8%) were hospitalized with CDI. The 1-year CDI incidence significantly increased in annual cohorts (ie, from 23.1; 95% CI, 12.8-41.8 per 1000 person-years in 2004 to 46.7; 95% CI, 35.0-62.3 per 1000 person-years in 2017; *P* = .001). *Clostridioides difficile* was associated with a 16.8% rate (n = 122) of 90-day mortality. In patients who underwent kidney transplant, CDI was typically late-onset (median interval, 2.2; IQR, 0.4-6.0 years) compared with recipients of other organs. Acute kidney injury requiring dialysis was significantly associated with short-term (adjusted odds ratio [aOR], 1.86; 95% CI, 1.07-3.26) and long-term (adjusted hazard ratio [aHR], 1.89; 95% CI, 1.29-2.78) mortality, and late-onset CDI was also significantly associated with a greater risk of short-term (aOR, 4.26; 95% CI, 2.51-7.22) and long-term (aHR, 2.49; 95% CI, 1.78-3.49) mortality.

**Conclusions and Relevance:**

In this study, increasing CDI trends in annual cohorts of SOT recipients were observed. Posttransplant CDI was associated with mortality, and late-onset CDI was associated with a greater risk of death than early-onset CDI. These findings suggest that preventive strategies should not be limited to the initial months following transplantation. Comprehensive therapeutic approaches targeting acute kidney injury risk factors in SOT recipients may reduce short- and long-term post-CDI mortality.

## Key Points

**Question:**

What are the incidence and outcomes associated with posttransplant *Clostridioides difficile* infection (CDI) in people who have undergone solid organ transplant?

**Findings:**

In this cohort study including 10 724 patients, posttransplant CDI was associated with a 90-day mortality incidence of 17%. Late-onset CDI was associated with a greater risk of short- and long-term mortality compared with early-onset CDI.

**Meaning:**

The findings of this large cohort of organ transplant recipients suggest a trend toward increasing risk of CDI over time.

## Teaser

This cohort study examines the incidence and outcomes associated with the development of *Clostridioides difficile* infection in individuals who have undergone solid organ transplant.

## Introduction

Patients who have undergone solid organ transplant (SOT) are at risk of *Clostridioides difficile* infection (CDI), which is associated with significant morbidity and mortality.^1,2,3^ A wide range of the cumulative CDI incidence has been reported in single-center studies^4,5^; however, SOT-related CDI incidence has been rarely estimated at the population level.^6^

Most studies have investigated early-onset CDI occurring within 90 days following transplant.^4,5,6,7^ However, the outcome of late-onset CDI has been rarely investigated.^4,5^ Although early-onset infectious complications may affect allograft function, late-onset infectious diseases may be associated with allograft loss and death.^8,9^ To our knowledge, post-CDI mortality has not been compared between patients who underwent SOT developing early- vs late-onset CDI.

SOT recipients are at risk of CDI complications.^10^ The frequency of colectomy in patients with SOT who develop CDI is more than 3 times higher than the rate of post-CDI colectomy in the general population.^11^ Complications of CDI are not limited to fulminant colitis. A recent study showed CDI considerably increases the risk of acute kidney injury (AKI) in patients without SOT.^12^ SOT recipients are at risk of AKI following infectious complications due to continuous exposure to nephrotoxic drugs, allograft rejection, and comorbidities.^13^ However, to our knowledge, the incidence and outcomes of AKI have not been investigated in patients with posttransplant CDI.

In this 15-year, population-based cohort study, we investigated the incidence and outcomes associated with posttransplant CDI among recipients of different organ allografts. Our findings may have implications for preventive strategies targeting SOT recipients who are at risk of CDI-associated complications and mortality.

## Methods

### Study Design

We undertook a population-based cohort study using Ontario administrative health care data held at ICES, a not-for-profit research institute in Ontario, Canada (eAppendix, eTable 1, and eTable 2 in the Supplement).^14^ Use of the data without informed consent was authorized under section 45 of Ontario’s Personal Health Information Protection Act and did not require research ethics board approval. Ontario has a universal, publicly funded, single-payer health insurance system that is administered by the provincial government. Data are reported herein in accordance with the Strengthening the Reporting of Observational Studies in Epidemiology (STROBE) guideline.^15^

We conducted this population-based study in Ontario, Canada’s most populous province (14.7 million in 2020). Approximately half of Canadian organ transplant procedures are performed in Ontario.^16^

### Study Population

We included all adults who received an SOT in Ontario between April 1, 2003, and December 31, 2017, with a maximum follow-up date of March 31, 2020. We excluded non-Ontario residents, individuals younger than 18 years, patients with no recorded date of birth or sex, those with a date of death on or before the date of organ transplant (the study cohort entry and index date), and those with a prior SOT (eFigure 1 in the Supplement). For each person, we studied the first organ transplant during the accrual period, allowing individuals to contribute multiple SOT events.

We considered the date of transplantation to be the cohort entry and index date for characterizing patient conditions and evaluating outcomes. Baseline characteristics included donor and recipient demographic characteristics, neighborhood income quintile, rural vs urban residence, comorbidities, and whether the organ was from a deceased vs living donor. We used a 365-day look-back window to determine the presence of pretransplant CDI, prior hospitalizations, emergency department visits, major abdominal procedures, nasogastric tube placement, and mechanical ventilation. We used a 5-year look-back period to identify comorbidities, including diabetes, cancer, inflammatory bowel diseases, chronic obstructive pulmonary disease, chronic kidney disease, and the Deyo-Charlson Comorbidity Index (eTable 3 in the Supplement).

The exposure comprised SOT categorized according to allograft type (ie, kidney, liver, kidney-pancreas, heart, lung, and multiorgan transplant). Owing to the small number of individuals in some subgroups, we created 3 transplant categories in some analyses: abdominal (ie, kidney, liver, and kidney-pancreas allografts), thoracic (ie, lung and heart allografts), and multiorgan transplant. Multiorgan transplant recipients included patients who received at least 2 organ allografts during the same hospitalization other than those who received a kidney-pancreas transplant.

### Study Outcomes

The primary outcome was hospital admission with a diagnosis of *C difficile* infection (*International Classification of Diseases, 10th Revision, Canadian* code A04.7), which has been reported to have high sensitivity (82.1%) and specificity (99.4%).^17^ We defined early-onset CDI as *C difficile* infections occurring during the index hospitalization or within 90 days of discharge. Episodes of CDI that occurred beyond this point were considered as late-onset CDI. Patients were censored on the date of death, loss of insurance coverage, or the end of the study period (March 31, 2020), whichever occurred first.

The secondary outcomes included all-cause death, intensive care unit (ICU) admission, AKI requiring dialysis, and fulminant CDI comprising any of the following: toxic megacolon, ileus, perforation, and colectomy. We determined the risk of short- vs long-term death. We defined short-term mortality as all-cause death occurring from the CDI hospitalization to 90 days postdischarge. Death occurring after this point was considered long-term mortality. For the outcome of AKI requiring dialysis, patients receiving long-term dialysis following transplant were excluded.

### Statistical Analysis

We determined baseline characteristics at the time of transplant as frequencies (percentages) for categorical variables and medians (IQRs) for continuous variables. We express incidence per 1000 person-years of observation (ie, patients with SOT and then recipients of different allografts).^18^ To evaluate trends in CDI incidence, we estimated the incidence for each annual SOT cohort using 1- and 3-year follow-up periods. We used the Cochran-Armitage test to compare trends in CDI incidence.

We estimated the cumulative probability of CDI (1- Kaplan-Meier) and 95% CI) overall and for each SOT type, using the Kaplan-Meier estimator, and compared the probability among SOT types, using the log rank test. We used Cox proportional hazards regression to identify patient characteristics independently associated with posttransplant CDI.

Next, we restricted the cohort to SOT recipients who developed CDI and determined the frequency of fulminant illness, AKI requiring acute dialysis, ICU admission, and mortality. Subgroup comparisons were conducted with χ^2^ tests.

We used logistic regression to quantify the association between short-term mortality and age, sex, Deyo-Charlson Comorbidity Index, SOT type (ie, thoracic vs abdominal transplant), early- vs late-onset CDI, fulminant CDI, ICU admission, and AKI requiring acute dialysis. We then used a Cox proportional hazards regression model to quantify the association between the same variables and long-term mortality, with patients censored on transplant of any organ type after the index hospitalization and the end of the study period (March 31, 2020). Statistical tests were 2-sided with the level of significance set at α = .05. All analyses were performed at ICES using SAS, version 9.4 (SAS Institute Inc).

## Results

### Patient Characteristics

A total of 12 192 patients received organ allografts in Ontario from April 1, 2003, to December 31, 2017. After exclusions, 10 724 patients were eligible for the study (mean [SD] age, 52.33 (12.94) years; 6901 [64.4%] men; 3823 [35.6%] women) (eAppendix, eTable 2, and eFigure 1 in the Supplement). Kidney transplant was the most common SOT type (6453 [60.2%]); multiorgan transplant was the least frequent (57 [0.5%]). The characteristics of SOT recipients varied according to allograft types (Table 1).

**Table 1.** Baseline Characteristics of Patients Who Received Solid Organ Transplant in Ontario

Variable	No. (%)						
	All SOT (n = 10 724)	Kidney (n = 6453)	Liver (n = 2117)	Kidney-pancreas (n = 452)	Heart (n = 523)	Lung (n = 1122)	Multiorgan (n = 57)
**Index year^a^**							
2003	316 (2.9)	206 (3.2)	56 (2.6)	10 (2.2)	18 (3.4)	26 (2.3)	0
2004	512 (4.8)	301 (4.7)	128 (6.0)	16 (3.5)	25 (4.8)	42 (3.7)	0
2005	530 (4.9)	315 (4.9)	112 (5.3)	23 (5.1)	29 (5.5)	49 (4.4)	8 (14)^b^
2006	620 (5.8)	379 (5.9)	129 (6.1)	21 (4.6)	28 (5.4)	61 (5.4)	
2007	663 (6.2)	403 (6.2)	135 (6.4)	24 (5.3)	28 (5.4)	71 (6.3)	
2008	631 (5.9)	387 (6.0)	130 (6.1)	24 (5.3)	28 (5.4)	60 (5.3)	
2009	721 (6.7)	477 (7.4)	127 (6.0)	27 (6.0)	26 (5.0)	63 (5.6)	14 (24.5)^b^
2010	669 (6.2)	415 (6.4)	130 (6.1)	25 (5.5)	29 (5.5)	66 (5.9)	
2011	714 (6.7)	429 (6.6)	146 (6.9)	30 (6.6)	28 (5.4)	80 (7.1)	
2012	758 (7.1)	469 (7.3)	151 (7.1)	37 (8.2)	34 (6.5)	64 (5.7)	
2013	741 (6.9)	427 (6.6)	132 (6.2)	36 (8.0)	45 (8.6)	96 (8.6)	
2014	857 (8.0)	504 (7.8)	171 (8.1)	41 (9.1)	37 (7.1)	92 (8.2)	12 (21.1)
2015	869 (8.1)	507 (7.9)	162 (7.7)	42 (9.3)	49 (9.4)	102 (9.1)	7 (12.3)
2016	1076 (10.0)	629 (9.7)	208 (9.8)	54 (11.9)	62 (11.9)	113 (10.1)	10 (17.5)
2017	1047 (9.8)	605 (9.4)	200 (9.4)	42 (9.3)	57 (10.9)	137 (12.2)	6 (10.5)
**Recipient**							
Age, y							
Median (IQR)	54 (44-62)	54 (43-63)	56 (49-61)	43 (37-50)	53 (43-60)	57 (45-63)	53 (48-59)
18-50	4197 (39.1)	2619 (40.6)	614 (29.0)	355 (78.5)	224 (42.8)	366 (32.6)	19 (33.3)
>51	6527 (60.9)	3834 (59.4)	1503 (71.0)	97 (21.5)	299 (57.2)	756 (67.4)	38 (66.7)
Sex							
Male	6901 (64.4)	4055 (62.8)	1483 (70.1)	290 (64.2)	392 (75.0)	646 (57.6)	35 (61.4)
Female	3823 (35.6)	2398 (37.2)	634 (29.9)	162 (35.8)	131 (25.0)	476 (42.2)	22 (38.6)
Income quintile^c^							
1	2343 (21.8)	1486 (23.0)	437 (20.6)	95 (21.0)	85 (16.3)	227 (20.2)	13 (22.8)
2	2137 (19.9)	1309 (20.3)	417 (19.7)	92 (20.4)	101 (19.3)	201 (17.9)	17 (29.8)
3	2121 (19.8)	1266 (19.6)	439 (20.7)^d^	100 (22.1)^d^	105 (20.1)^d^	212 (18.9)	9 (15.8)
4	2124 (19.8)	1261 (19.5)	426 (20.1)	84 (18.6)	113 (21.6)	229 (20.4)	11 (19.3)
5	1967 (18.3)	1117 (17.3)	398 (18.8)	81 (17.9)	119 (22.8)	245 (21.8)	7 (12.3)
Residence							
Rural	1146 (10.7)	671 (10.4)	212 (10.0)	50 (11.1)	63 (12.0)	143 (12.7)	7 (12.3)
**Donor**							
Age, median (IQR)	47 (34-57)	48 (37-57)	47 (31-58)	26 (20-39)	38 (24-49)	49 (33-59)	44 (34-52)
Sex							
Male	5320 (49.6)	3274 (50.7)	1072 (50.6)	249 (55.1)	269 (51.4)	425 (37.9)	31 (54.4)
Living donor	2940 (27.4)	2429 (37.6)	511 (24.1)	0 (0.0)	0 (0.0)	0 (0.0)	0
**Blood type**							
Donor							
A	3547 (33.1)	2209 (34.2)	707 (33.4)	153 (33.8)	151 (28.9)	303 (27.0)	25 (4)^e^
AB	284 (2.6)	199 (3.1)	58 (2.7)	9 (2.0)	7 (1.3)	8 (0.7)	13 (22.8)^b^
B	1186 (11.1)	752 (11.7)	246 (11.6)	48 (10.6)	38 (7.3)	92 (8.2)	
O	4905 (45.7)	3162 (49.0)	942 (44.5)	199 (44.0)	200 (38.2)	383 (34.1)	19 (33.3)
Recipient^c^							
A	4104 (38.3)	2385 (37.0)	832 (39.3)^e^	167 (36.9)	226 (43.2)	474 (42.2)^e^	26 (45.6)
AB	543 (5.1)	332 (5.1)	120 (5.7)	13 (2.9)	30 (5.7)	45 (4.0)	13 (22.8)^b^
B	1540 (14.4)	944 (14.6)	307 (14.5)	61 (13.5)	81 (15.5)	137 (12.2)	
O	4394 (41.0)	2672 (41.4)	858 (40.5)	194 (42.9)	186 (35.6)	466 (41.5)	18 (31.6)
**Graft function**							
Delayed graft function^f^	NA	1685 (26.1)	NA	NA	NA	NA	NA
Length of index transplant episode, median (IQR), d	10 (7-18)	8 (6-11)	15 (10-33)	11 (9-15)	28 (15-58)	25 (16-51)	25 (13-53)
**Comorbidities (look back 5 y)**							
Diabetes	4305 (40.1)	2418 (37.5)	856 (40.4)	452 (100.0)	227 (43.4)	329 (29.3)	23 (40.4)
Deyo-Charlson comorbidity index							
Median (IQR)	3 (2-4)	2 (2-4)	4 (3-5)	4 (4-5)	2 (2-4)	2 (2-2)	5 (3-7)
2	5215 (48.6)	3648 (56.5)	218 (10.3)	51 (11.3)	271 (51.8)	1017 (90.6)	10 (17.5)
3	1589 (14.8)	662 (10.3)	751 (35.5)	274 (60.6)^b^	94 (18.0)	70 (6.2)	11 (19.3)^b^
4	1974 (18.4)	1238 (19.2)	368 (17.4)		75 (14.3)	20 (1.8)	
≥5	1946 (18.1)	905 (14.0)	780 (36.8)	127 (28.1)	83 (15.9)	15 (1.3)	36 (63.2)
Inflammatory bowel disease	213 (2.0)	77 (1.2)	124 (5.9)	≤5^b^	≤5^b^	≤5^b^	≤5^b^
COPD	2003 (18.7)	759 (11.8)	320 (15.1)	25 (5.5)	108 (20.7)	779 (69.4)	12 (21.1)
Cancer	3882 (36.2)	1935 (30.0)	1299 (61.4)	120 (26.5)	108 (20.7)	390 (34.8)	30 (52.6)
Chronic kidney disease	7633 (71.2)	6447 (99.9)	450 (21.3)	452 (100.0)	168 (32.1)	68 (6.1)	48 (84.2)
**Comorbidities (look back, 1 y)**							
No. of hospital admissions							
Median (IQR)	1 (0-3)	1 (0-2)	3 (2-6)	2 (1-3)	3 (2-5)	2 (1-4)	3 (2-5)
0	2798 (26.1)	2497 (38.7)	75 (3.5)	99 (21.9)	26 (5.0)	96 (8.6)	6 (10.5)
1-2	4684 (43.7)	3022 (46.8)	726 (34.3)	214 (47.3)	159 (30.4)	545 (48.6)	18 (31.6)
≥3	3242 (30.2)	934 (14.5)	1316 (62.2)	139 (30.8)	338 (64.6)	481 (42.9)	33 (57.9)
No. of ED visits							
Median (IQR)	1 (0-2)	0 (0-2)	2 (1-5)	1 (0-3)	2 (1-4)	1 (0-3)	2 (1-6)
0	4326 (40.3)	3243 (50.3)	489 (23.1)	144 (31.9)	95 (18.2)	344 (30.7)	11 (19.3)
1-2	3791 (35.4)	2330 (36.1)	615 (29.1)	178 (39.4)	187 (35.8)	463 (41.3)	18 (31.6)
≥3	2607 (24.3)	880 (13.6)	1013 (47.9)	130 (28.8)	241 (46.1)	315 (28.1)	28 (49.1)
No. of hospital admissions or ED visits							
Median (IQR)	2 (1-5)	2 (0-3)	6 (3-11)	3 (1-5)	6 (4-9)	4 (2-6)	5 (2-12)
0	1831 (17.1)	1668 (25.8)	37 (1.7)	47 (10.4)	15 (2.9)	62 (5.5)	15 (26.3)^b^
1-2	3592 (33.5)	2603 (40.3)	425 (20.1)	156 (34.5)	68 (13.0)	327 (29.1)	
≥3	5301 (49.4)	2182 (33.8)	1655 (78.2)	249 (55.1)	440 (84.1)	733 (65.3)	42 (73.7)
Pretransplant CDI	93 (0.9)	25 (0.4)	46 (2.2)	≤5^b^	≤5^b^	11 (1.0)	≤5^b^
Major abdominal surgeries	650 (6.1)	342 (5.3)	236 (11.1)	23 (5.1)	22 (4.2)	23 (2.0)	≤5^b^
Mechanical ventilation	422 (3.9)	92 (1.4)	136 (6.4)	13 (2.9)	99 (18.9)	76 (6.8)	6 (10.5)

Abbreviations: CDI, *Clostridioides difficile* infection; COPD, chronic obstructive pulmonary disease; ED, emergency department; SOT, solid organ transplant.

^a^ Index year: year of transplant.

^b^ In accordance with ICES privacy policies, cell sizes less than or equal to 5 cannot be reported.

^c^ Missing information: donor type, 770 (7.2%); blood type in recipient, 143 (1.3%); blood type in donor, 802 (7.5%); and income quantile, 32 (0.3%).

^d^ Missing information in this subgroup was less than or equal to 5 and imputed in income quantile 3.

^e^ Missing information in this subgroup was less than or equal to 5 and imputed in blood group A.

^f^ This variable was only studied in kidney transplant recipients.

### CDI Incidence

**Figure 1.**
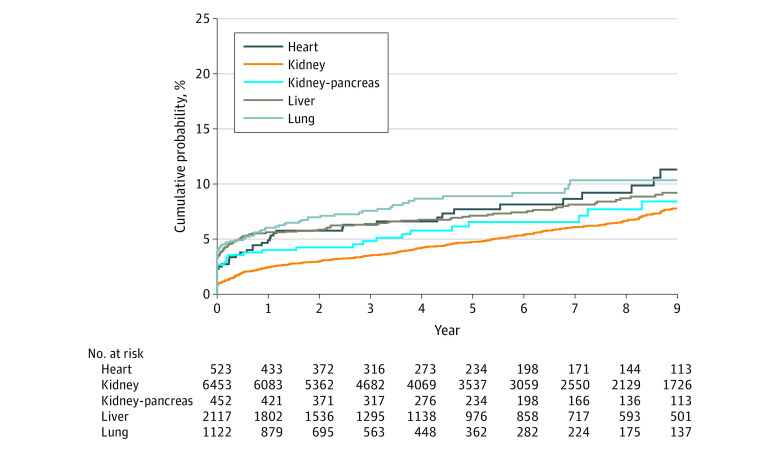
Cumulative Probability of *Clostridioides difficile* Infection According to Solid Organ Transplant Type

The median follow-up time was 5.0 (IQR, 2.3-8.8) years, resulting in 61 987 person-years of follow-up. Overall, we identified 726 CDI hospitalizations (6.8%), with 275 early-onset (37.9%) occurring within 90 days of transplantation and 451 late-onset (62.1%) occurring thereafter. The overall CDI incidence rate was 11.7 (95% CI, 10.9-12.6) per 1000 person-years. Table 2 provides the CDI incidence by SOT type. Although CDI frequently occurred within the first year after transplant, recipients of SOT other than the kidney (median interval since transplant, 0.9; IQR, 0.0-4.6 years), CDI was typically a late-onset complication in kidney allograft recipients (median interval since transplant, 2.2; IQR, 0.4-6.0 years). The highest incidence rate was in patients who underwent multiorgan transplant (45.3; 95% CI, 23.6-87.1 per 1000 person-years) followed by lung transplant (20.6; 95% CI, 16.8-25.2 per 1000 person-years). Kidney allograft recipients had the lowest CDI incidence (9.6; 95% CI, 8.7-10.6 per 1000 person-years). Development of CDI in patients who received kidney transplant was typically late-onset (median interval between transplant and CDI, 2.2; IQR, 0.4-6.0 years). Figure 1 shows the cumulative probability of CDI (1-KM) was considerably different in recipients of different allografts. Although CDI probability continuously increased in all SOT types (eAppendix, eFigure 2 in the Supplement), the risk of CDI differed among SOT types (*P*<.001).

**Table 2.** CDI Incidence Rate, Cumulative Incidence, and Interval Between Transplant Date and CDI

SOT type	No.	Total follow up, person-years	CDI		HR (95% CI)	Cumulative CDI incidence (95% CI), %			Onset, median (IQR), y^a^
			No. (%)	Incidence (95% CI), 1000 person-years^a^		1 y	5 y	10 y	
Total	10724	61 987.1	726 (6.8)	11.7 (10.9-12.6)		3.6 (3.3-4.0)	5.6 (5.1-6.0)	8.3 (7.7-9.0)	0.9 (0.0-4.6)
Kidney	6453	40 221.5	386 (6.0)	9.6 (8.7-10.6)	1 [Reference]	2.4 (2.1-2.8)	4.5 (4.0-5.1)	7.7 (6.9-8.6)	2.2 (0.4-6.0)
Liver	2117	11 579.5	162 (7.7)	14.0 (12.0-16.3)	1.400 (1.165-1.682)	5.5 (4.6-6.5)	6.7 (5.7-7.9)	8.5 (7.2-9.9)	0.2 (0.0-2.2)
Kidney-pancreas	452	2716.9	33 (7.3)	12.1 (8.6-17.1)	1.254 (0.879-1.789)	4.0 (2.4-6.1)	6.3 (4.2-9.0)	9.3 (6.2-13.1)	0.7 (0.0-4.6)
Heart	523	2754.4	43 (8.2)	15.6 (11.6-21.0)	1.546 (1.128-2.118)	4.6 (3.0-6.6)	6.9 (4.9-9.5)	10.1 (7.2-13.7)	0.7 (0.0-4.4)
Lung	1122	4516.3	93 (8.3)	20.6 (16.8-25.2)	1.821 (1.452-2.283)	5.8 (4.5-7.3)	7.9 (6.3-9.6)	9.4 (7.6-11.6)	0.1 (0.0-1.8)
Multiorgan	57	198.6	9 (15.8)	45.3 (23.6-87.1)	3.860 (1.958-7.611)	12.3 (5.4-22.3)	14.1 (6.5-24.4)	23.6 (7.2-45.1)	0.0 (0.0-1.1)

Abbreviations: CDI, *Clostridioides difficile* infection; HR, hazard ratio; SOT, solid organ transplant.

^a^ Interval between hospital admission for transplant and CDI.

### Trends in CDI Incidence

**Figure 2.**
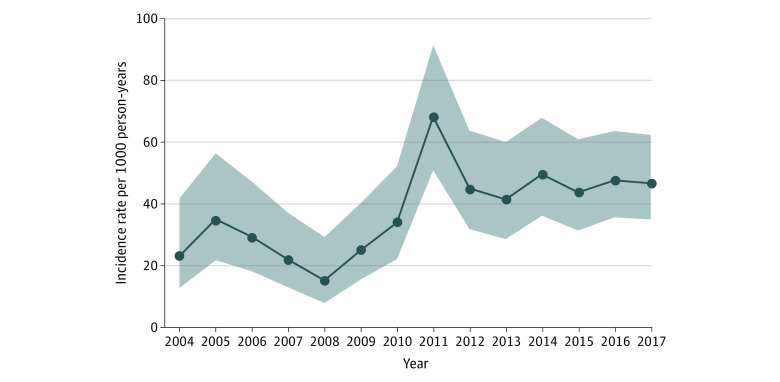
Trend of 1-Year *Clostridioides difficile* infection Incidence in Annual Cohorts of Solid Organ Transplant Recipients

Figure 2 and eTable 4 in the Supplement provide the CDI incidence in annual cohorts of SOT recipients at 1- and 3-year follow-up. After an abrupt increase of incidence from 34.0 per 1000 person-years (95% CI, 22.2-52.1 per 1000 person-years) in 2010 to 68.2 per 1000 person-years (95% CI, 50.9-91.4 per 1000 person-years) in 2011, the CDI incidence remained elevated and did not return to the range observed during 2003-2010 (*P*<.001). eTable 5 in the Supplement provides the 1-year posttransplant CDI incidence in annual cohorts of kidney, liver, and thoracic organ allograft recipients from 2011 to 2017. A Cochran-Armitage test showed the 3-year CDI incidence among patients who received transplants in 2011-2015 was greater than among those who received transplants in 2003-2010 (*P*<.001).

### Patient Characteristics Associated With CDI

We used a series of Cox proportional hazards regression models to investigate the association between potential baseline contributing factors and posttransplant CDI (eAppendix; eTable 6 in the Supplement). We found an association between age at the time of transplant and posttransplant CDI (hazard ratio [HR], 1.14 for each 10-year increase in age; 95% CI, 1.07-1.22; *P* < .001). Patients who received an SOT other than kidney were at a significantly higher risk of CDI compared with those who received a kidney transplant. However, in kidney allograft recipients, delayed graft function was associated with a greater risk of CDI (HR, 1.6; 95% CI, 1.3-2.0). Comorbidities, such as diabetes (HR, 1.6; 95% CI, 1.4-1.9), inflammatory bowel disease (HR, 1.7; 95% CI, 1.1-2.5), chronic obstructive pulmonary disease (HR, 1.5; 95% CI, 1.2-1.8), and cancer (HR, 1.4; 95% CI, 1.2-1.6), were significantly associated with a higher risk of CDI.

### Factors Associated With Mortality

Overall, 43 patients (5.9%) developed fulminant CDI (ie, ileus, toxic megacolon, and intestinal perforation). Of these, 26 patients (60.4%) required colectomy. In multivariable analysis, the association between fulminant CDI (5.9%) and mortality was not significant (aOR, 1.14; 95% CI, 0.51-2.55). The frequency of fulminant CDI was not significantly different in recipients of thoracic vs abdominal organs (9 of 136 [6.6%] vs 34 of 581 [5.9%]; *P* = .73). Post-CDI colectomy was not associated with a risk for short-term mortality (7 of 26 [26.9%]) vs patients who did not undergo colectomy (115 of 700 [16.4%]) (*P* = .16).

Overall, 368 of 726 patients (50.7%) who had undergone SOT and developed CDI were admitted to the ICU during the CDI hospitalization. Patients who were admitted to the ICU were at a greater risk of post-CDI short-term mortality compared with patients who did not require ICU admission (84 of 368 [22.8%] vs 38 of 358 [10.6%]; *P* = .001). Intensive care unit admission was more frequent among thoracic (92 of 136 [67.6%]) than among abdominal (267 of 581 [46.0%]) SOT recipients with CDI vs (*P* = .001].

In total, 97 patients with CDI (13.4%) developed AKI requiring acute dialysis. The frequency of acute dialysis in thoracic SOT recipients was significantly greater than in abdominal allograft recipients (31 of 136 [22.8%] vs 66 of 581 [11.4%]; *P* = .001). Patients who received a heart transplant had the highest frequency of dialysis-requiring AKI (25.6%) followed by lung allograft recipients (21.5%). In contrast, the frequency of acute dialysis was considerably lower in all patients who received abdominal organ transplants, including liver (9.9%), kidney (11.9%), and kidney-pancreas (12.1%). There was an association between patients with vs without the need for acute dialysis and short-term mortality (28 of 97 [28.9%] vs 94 of 629 [14.9%]; *P* = .001). Acute dialysis was associated with a greater risk of short-term mortality in both abdominal (18 of 66 [27.3%] vs 76 of 515 [14.8%]; *P* = .009) and thoracic (10 of 31 [32.3%] vs 16 of 105 [15.2]; *P* = .03) SOT recipients.

### Short-term Mortality Following CDI

The short-term (90-day) mortality rate was 16.8% (122 of 726). Patients who received lung transplants had the largest short-term mortality rate compared with recipients of other SOT with CDI (21 of 93 [22.6%] vs 101 of 633 [15.9%]; *P* = .11). Short-term mortality was not significantly different between abdominal and thoracic transplant recipients (94 of 581 [16.2%] vs 26 of 136 [19.1%]; *P* = .41).

Late-onset CDI was associated with a significantly greater short-term mortality than early-onset CDI (97 of 451 [21.5%] vs 25 of 275 [9.1%]; *P* = .001). In a logistic regression model, age (adjusted odds ratio [aOR], 1.02; 95% CI, 1.01-1.04), Deyo-Charlson comorbidity index (aOR, 1.11; 95% CI, 1.00-1.22), AKI requiring dialysis (aOR, 1.86; 95% CI, 1.07-3.26), and ICU admission (aOR, 3.77; 95% CI, 2.33-6.07) were associated with an increased risk of 90-day mortality. Late-onset CDI was associated with a higher odds of 90-day mortality following CDI hospitalization (aOR, 4.26; 95% CI, 2.51-7.22) (Table 3).

**Table 3.** Risk Factors for Short-term vs Long-term Mortality^a^

Covariate	Risk of mortality	
	Short-term, OR (95% CI)^b^	Long-term, HR (95% CI)^c^
Thoracic vs abdominal SOT	1.55 (0.89-2.68)	2.18 (1.55-3.06)
Age based on CDI event	1.02 (1.01-1.04)	1.03 (1.02-1.05)
Sex, recipient (female)	0.82 (0.52-1.27)	1.11 (0.84-1.47)
Deyo-Charlson score based on CDI event	1.11 (1.00-1.22)	1.180 (1.10-1.26)
Acute dialysis	1.86 (1.07-3.26)	1.90 (1.29-2.78)
Fulminant colitis	1.14 (0.51-2.55)	1.17 (0.68-2.02)
ICU admission	3.77 (2.34-6.08)	1.21 (0.88-1.65)
Late vs early CDI	4.26 (2.51-7.22)	2.49 (1.78-3.49)

Abbreviations: CDI, *Clostridioides difficile* infection; HR, hazard ratio; ICU, intensive care unit; OR, odds ratio.

^a^ Short-term mortality defined as all-cause death occurring from the CDI hospitalization to 90 days post-discharge. Death occurring after this point was considered long-term mortality.

^b^ Logistic regression analysis.

^c^ Cox proportional hazards regression analysis.

### Long-term Mortality Following CDI

In Cox proportional hazards regression analysis, thoracic vs abdominal SOT (aHR, 2.18; 95% CI, 1.55-3.06), age (aHR, 1.03; 95% CI, 1.02-1.05), Deyo-Charlson comorbidity index at the time of CDI (aHR, 1.18; 95% CI, 1.10-1.26), and AKI requiring dialysis (aHR, 1.90; 95% CI, 1.29-2.78) were associated with an increased risk of longer-term mortality following CDI (Table 3). As with short-term mortality, late-onset CDI was associated with a relatively greater risk of longer-term death following CDI (aHR, 2.49; 95% CI, 1.78-3.49).

## Discussion

In the absence of population-based studies, estimation of posttransplant CDI incidence has been limited to single-center studies providing wide variation in cumulative CDI incidence.^2,19^ Our study, which is, to our knowledge, the largest cohort of SOT recipients including 10 724 patients and 61 987 person-years of follow-up, estimated a CDI incidence of 11.7 (95% CI, 10.9-12.6) per 1000 person-years. We observed a trend toward increasing risk of posttransplant CDI over time. Recent surveillance data showed an increasing CDI incidence trend in the US general population in 2011 followed by a relative plateau in 2012 and thereafter.^20^The increasing CDI incidence in 2011-2012 could have been due to the fact that 52% of microbiology laboratories used nucleic acid amplification testing, which is more sensitive than other test types.^20^ Nucleic acid amplification tests, compared with toxin enzyme immunoassay, could increase the CDI diagnosis by as much as 67% owing to greater sensitivity.^21^ A relatively greater incidence of CDI in Ontario in 2011 may have been associated with use of nucleic acid amplification testing, although the Provincial Infectious Diseases Advisory Committee in Ontario recommended using the nucleic acid amplification test in 2013.^22,23^ Pereira et al^24^ has recently reported on an increasing CDI trend in the general population of Ontario, but this pattern did not continue beyond 2012. Thus, the increasing trend of CDI in patients who undergo SOT requires focused attention on preventive strategies.

In this population-based cohort, SOT recipients were at considerable risk of post-CDI mortality. Overall, short-term mortality was 19.1% in thoracic SOT recipients and 16.2% in abdominal SOT recipients. The greatest short-term mortality was in patients who received a lung transplant (22.6%), which supports previous single-center data.^5^ Acute kidney injury requiring dialysis was associated with a significant risk of mortality (aOR, 1.86; 95% CI, 1.07-3.26). Generally, patients who undergo SOT are susceptible to AKI because of several factors, such as continuous exposure to nephrotoxic drugs, infectious complications, allograft rejection, and comorbidities.^13,25^ Acute kidney injury is an independent predictor of CDI severity^26^ and may occur regardless of fulminant CDI.^27^ Patients with CDI may develop AKI because of intrinsic kidney insult; direct toxin-associated kidney disease; and factors that occur before AKI, such as decreased organ perfusion,^28^ protein-losing enteropathy leading to reduced oncotic pressure,^29^ and immunoglobulin A nephropathy.^30^ We noted that dialysis-requiring AKI is an independent risk factor for short-term mortality. The association between AKI and long-term mortality remained significant in Cox proportional hazards regression analysis. Experimental data noted an association between AKI and tissue injury in different organs, such as inflammatory and functional changes in the brain, pulmonary vascular integrity loss, cardiac tissue apoptosis, and severe fibrotic changes in the kidneys.^31,32^ Acute kidney injury has been reported to increase the long-term risk of cardiovascular events.^33^ Thus, the longstanding association between AKI and mortality appears to be biologically plausible.

Recipients of SOT with CDI are more likely to be admitted to the ICU compared with patients who have not received an SOT.^34^ Patients who were admitted to the ICU were at greater risk of short-term mortality. However, this risk did not remain significant in patients who survived 90 days following the CDI hospitalization.

Timing of the onset of CDI was an important factor in CDI outcome. Most studies only investigated early-onset CDI and their findings were likely affected by incomplete follow-up.^6,7,35^ In our study, over 60% of CDI hospitalizations following transplant were late onset. Although CDI frequently occurred within the first year after transplant, recipients of SOT other than the kidney (median interval since transplant, 0.9; IQR, 0.0-4.6 years), CDI was typically a late-onset complication in kidney allograft recipients (median interval since transplant, 2.2; IQR, 0.4-6.0 years). Late- vs early-onset CDI was associated with a significantly greater risk of short- and long-term mortality. In the first 3 months after transplant, infectious complications are more likely secondary to transplant procedure.^36^ However, infectious complications occurring beyond 3 months following transplant may be associated with graft dysfunction, late-onset allograft rejection, and cumulative immunosuppression.^9,37^ To our knowledge, ours is the first study to quantify the risk of death in patients who have undergone SOT and developed CDI early vs later after transplant.

The frequency of fulminant CDI in our cohort was considerably greater than previous estimates in the general population.^38^ In multivariable analysis, the association between fulminant CDI (5.9%) and mortality was not significant (aOR, 1.14; 95% CI, 0.51-2.55). This finding may be associated with 60% of the patients with fulminant CDI undergoing colectomy. Our finding is consistent with previous studies showing colectomy may be life-saving in patients with fulminant CDI.^39,40,41^ Prospective studies are required to estimate postcolectomy mortality.

### Limitations

This study has limitations. We did not have access to information on immunosuppressive regimens and antibiotic therapies at an individual level. The present study was conducted with a primary focus on CDI outcomes rather than risk factors. The outcomes of interest did not include mild CDI episodes that were managed in outpatient settings. This cohort was restricted to transplant centers across Ontario, which may affect the generalizability of our findings. However, within the accrual window of this cohort, 40% to 50% of SOT operations in Canada have been done in Ontario.^16^

## Conclusions

In this study, we observed increasing CDI trends in annual cohorts of SOT recipients. Posttransplant CDI was associated with considerable mortality. Although CDI was an early-onset disease in non–kidney allograft recipients, patients who underwent kidney transplant typically experienced late-onset CDI. Late- vs early-onset CDI was associated with a greater risk of death. Acute kidney injury was also associated with an increased risk of short- and long-term mortality following CDI. Acute kidney injury preventive measures, such as adequate fluid repletion, avoidance of hypotension in critically ill SOT recipients, readjustment of nephrotoxic medications based on drug levels, and close kidney function monitoring, should be considered in the management of CDI. *Clostridioides difficile* infection is potentially preventable through risk reduction strategies. Further studies are required to reduce CDI incidence and related complications in SOT recipients.

## Notes

Supplement. **eAppendix.** Data Sources **eTable 1.** Data Sources Used in the Study **eTable 2.** Data Sources Used for Inclusion and Exclusion Criteria **eTable 3.** Variables **eTable 4.** CDI Incidence in Annual Cohorts of SOT Recipients 1 Year and 3 Years Following Transplant **eTable 5.** 1-Year Posttransplant CDI Incidence in Annual Cohorts of Renal, Liver, and Thoracic Organ Allograft Recipients From 2011 to 201 **eTable 6.** Baseline Contributing Factors to Posttransplant CDI
eReference
**eFigure 1.** Study Flowchart **eFigure 2.** Cumulative Probability of CDI for All SOT Recipients
