# Supplementary material for: Incidence and Outcomes Associated With Clostridioides difficile Infection in Solid Organ Transplant Recipients
Source: JAMA Netw Open. 2021 Dec 29;4(12):e2141089. doi: 10.1001/jamanetworkopen.2021.41089 (PMC8717111; doi:10.1001/jamanetworkopen.2021.41089)
Supplement: Supplement. — eAppendix. Data Sources eTable 1. Data Sources Used in the Study eTable 2. Data Sources Used for Inclusion and Exclusion Criteria eTable 3. Variables eTable 4. CDI Incidence in Annual Cohorts of SOT Recipients 1 Year and 3 Years Following Transplant eTable 5. 1-Year Posttransplant CDI Incidence in Annual Cohorts of Renal, Liver, and Thoracic Organ Allograft Recipients From 2011 to 201 eTable 6. Baseline Contributing Factors to Posttransplant CDI eReference eFigure 1. Study Flowchart eFigure 2. Cumulative Probability of CDI for All SOT Recipients [file jamanetwopen-e2141089-s001.pdf]

Supplementary Online Content

Hosseini-Moghaddam SM, Luo B, Bota SE, et al. Incidence and outcomes associated with *Clostridioides difficile* infection in solid organ transplant recipients. *JAMA Netw Open*. 2021;4(12):e2141089. doi:10.1001/jamanetworkopen.2021.41089

eAppendix. Data Sources

eTable 1. Data Sources Used in the Study

eTable 2. Data Sources Used for Inclusion and Exclusion Criteria

eTable 3. Variables

eTable 4. CDI Incidence in Annual Cohorts of SOT Recipients 1 Year and 3 Years Following Transplant

eTable 5. 1-Year Posttransplant CDI Incidence in Annual Cohorts of Renal, Liver, and Thoracic Organ Allograft Recipients From 2011 to 2017

eTable 6. Baseline Contributing Factors to Posttransplant CDI

eReference

eFigure 1. Study Flowchart

eFigure 2. Cumulative Probability of CDI for All SOT Recipients

This supplementary material has been provided by the authors to give readers additional information about their work.

© 2021 Hosseini-Moghaddam SM, et al. *JAMA Network Open.*

eAppendix. Data Sources

Using unique encoded identifiers, we linked multiple datasets at ICES. We used the Canadian Organ Replacement Register (CORR) to ascertain a cohort of SOT recipients[1]. We linked CORR data to the Canadian Institute for Health Information Discharge Abstract Database (CIHI-DAD) to identify hospital admissions in SOT recipients using International Classification of Diseases, 10th Revision, Canadian Modification (ICD-10-CA) diagnosis codes. We used the Registered Persons Database (RPDB) for demographic information and vital status. Information from multiple other databases such as Ontario Health Insurance Plan (OHIP) were linked at the individual level to derive baseline characteristics and covariates as presented in eTables 2-4.

© 2021 Hosseini-Moghaddam SM, et al. *JAMA Network Open.*

eTable 1. Data Sources Used in the Study

Source	Full Name	Description
CIHI DAD	Canadian Institute for Health Information-Discharge Abstract Database	The CIHI DAD database contains summarized hospital discharge information for individuals receiving inpatient care in a non-mental health designated bed. Each record contains the patient identifier, the date of admission and discharge, clinical details of the hospitalization (including diagnoses made and procedures received).
OHIP	Ontario Health Insurance Plan Claims Database	The OHIP database contains Ontario physician claims from inpatient, outpatient, and long-term care settings. Each record identifies the physician, the patient, the diagnosis responsible for the claim (which follows the coding scheme in the International Classification of Diseases, 9th edition), the service provided, and the date on which the service was provided.
CCI-CIHI	Canadian Classification of Health Interventions-Canadian Institute for Health Information	CCI-CIHI contains information related to medical or surgical interventions.
CCP	Canadian Classification of diagnostic, therapeutic and surgical Procedures	CCP contains information related to diagnostic, therapeutic, and surgical procedures using ICD-9 codes. CCP is divided into two parts: the tabular list and the alphabetic index.
CPDB	Corporate Providers Database	The CPDB contains information about all physician and some non-physician (such as chiropractors, physiotherapists, and optometrist) providers funded by the Ontario Ministry of Health. The information received includes demographics, eligibility for payment, and affiliation to hospital, specialty, and practice location.
NACRS	National Ambulatory Care Reporting System	NACRS contains information on patient visits to hospital and community-based ambulatory and emergency care. Each record contains the patient identifier, date of registration, and clinical details of the visit.
IPDB	Institute for Clinical Evaluative Sciences Physician Database	The IPDB contains yearly information about all physicians in Ontario, including physician demographic characteristics, specialty (functional and certified), location, and measures of physician activity (billings, workload, types of services provided). The IPDB comprises information from the CPDB, the Ontario Physician Human Resource Data Centre database, and the OHIP database of physician billings.
RPDB	Registered Persons Database	The RPDB contains demographic information such as age, sex, health insurance eligibility and death information for anyone who has received Ontario health care coverage. It contains postal code information that is linkable to other geographic information such as dissemination areas which are needed to link to the Ontario Marginalization Index and other census-derived, neighborhood-level information.
CORR	Canadian Organ Replacement Register	CORR is a pan-Canadian information system managed by CIHI. CORR contains data from all hospital dialysis programs, transplant programs, organ procurement organizations (OPOs)

© 2021 Hosseini-Moghaddam SM, et al. *JAMA Network Open.*

		and independent health facilities to track patients from their first treatment for end stage organ failure including dialysis or organ transplantation to their death.
COPD	Ontario Chronic Obstructive Pulmonary Disease	The Ontario COPD cohort contains all Ontario COPD patients identified since 1991.

© 2021 Hosseini-Moghaddam SM, et al. *JAMA Network Open.*

eTable 2. Data Sources Used for inclusion and exclusion criteria

	Database	Code
**Inclusion Criteria**		
Recipients of organ allograft including liver, kidney, lung, pancreas, heart, and multiorgan	CORR	RECIPIENT_TREATMENT<br>TRANSPLANTED_ORGAN_TYPE_CODE 1-3
Records in DAD	CIHI-DAD	Admdate<br>ddate
**Exclusion criteria**		
Missing or invalid IKN	RPDB	
Missing age	RPDB	rpdbdemo
Missing sex	RPDB	rpdbdemo
Death on or before the index date	RPDB	RPDB
Non-Ontario residents	RPDB	rpdbpstl
Invalid age (>105)	RPDB	rpdbdemo
Patients with any organ transplant before the index date	CORR, DAD OHIP, CCP, CCI	RECIPIENT_TREATMENT<br>TREATMENT_CODE<br>OHIP, CCP and CCI codes used to identify patients received organ transplant before 2002

© 2021 Hosseini-Moghaddam SM, et al. *JAMA Network Open.*

eTable 3. Variables

Variable	Database	Code
**Baseline**		
Age, recipient	RPDB	
Sex, recipient	RPDB	
Blood type, recipient	CORR	
Age, donor	CORR	Donor dataset
Sex, donor	CORR	Donor dataset
Blood type, donor	CORR	Donor dataset
Donor status	CORR	Donor dataset
Delayed graft function	OHIP<br>DAD	Acute dialysis in the first week of transplant
Length of stay for index hospitalization	DAD	
Income quantile	RPDB	Quantiles 1-5
Residency status	RPDB	Rural vs. urban
Number of hospitalizations	DAD	Source: all<br>Institution types: Acute care
Number of emergency department visits	NACRS	
Cancer	DAD<br>OHIP	DAD (ICD-9, ICD-10), OHIP
Diabetes mellitus	ODD (ICES derived cohort)	
Chronic obstructive pulmonary disease	COPD (validated ICES-Derived cohort)	
Chronic kidney disease	OHIP<br>DAD	ICD-10
Inflammatory bowel disease	OCCC	Ontario Crohn's and Colitis Cohort dataset
Hospital admission or emergency room visit	CIHI-DAD<br>NACRS<br>OHIP	
Charlson Comorbidity Index (CCI)	DAD	
Pre-transplant CDI	DAD	ICD-10-CA: A04.7
Major abdominal surgeries	OHIP	Diagnostic fee codes
NG tube placement	DAD<br>CCI	
Mechanical ventilation	DAD<br>CCI	

© 2021 Hosseini-Moghaddam SM, et al. *JAMA Network Open.*

**Exposure**		
Organ transplant	CORR	
**Outcomes**		
CDI	DAD	ICD-10-CA: A04.7
All-cause death	RPDB<br>CORR	
Acute dialysis	OHIP	
ICU admission	SCU<br>CCP<br>OHIP<br>CCI	
Colectomy	DAD, CCI	
Intestinal complication	DAD	ICD-10 codes: Ileus, toxic megacolon
Organ Transplant	CORR	

© 2021 Hosseini-Moghaddam SM, et al. *JAMA Network Open.*

eTable 4. CDI incidence in annual cohorts of SOT recipients 1 year and 3 years following transplant

Year	1-year follow up			3-year follow up		
	CDI	Person-year	Incidence rate per 1000 person-year (95% CI)	CDI	Person-year	Incidence rate per 1000 person-year (95% CI)
2004	11	476	23.1(12.8-41.8)	18	1380	13.0(8.2-20.7)
2005	17	485	35.0 (21.8-56.4)	21	1401	15.0 (9.8-23.0)
2006	17	580	29.3 (18.2-47.2)	20	1665	12.0 (7.7-18.6)
2007	14	638	22.0 (13.0-37.1)	18	1830	9.8 (6.2-15.6)
2008	9	591	15.2 (7.9-29.3)	14	1703	8.2 (4.9-13.9)
2009	17	680	25.0 (15.5-40.2)	23	1981	11.6 (7.7-17.5)
2010	21	618	34.0(22.2-52.1)	26	1767	14.7 (10.0-21.6)
2011	45	660	68.2 (50.9-91.4)	53	1894	28.0 (21.4-36.6)
2012	32	710	45.1 (31.9-63.7)	39	2040	19.1 (14.0-26.2)
2013	28	676	41.4 (28.6-60.0)	36	1936	18.6 (13.4-25.8)
2014	39	787	49.6 (36.2-67.9)	48	2247	21.4 (16.1-28.4)
2015	35	800	43.7 (31.4-60.9)	45	2300	19.6 (14.6-26.2)
2016	46	966	47.6 (35.7-63.6)			
2017	46	985	46.7 (35.0-62.3)			

© 2021 Hosseini-Moghaddam SM, et al. *JAMA Network Open.*

eTable 5. 1-year post-transplant CDI incidence in annual cohorts of renal, liver, and thoracic organ allograft recipients from 2011 to 2017

Year	Number of CDI events	Person-year follow up	Incidence rate per 1000 Person-year (95% CI)
Renal transplants			
2011	21	442	47.5 (31.0-72.9)
2012	16	485	33.0 (20.2-53.9)
2013	11	447	24.6 (13.6-44.5)
2014	19	516	36.9 (23.5-57.8)
2015	17	534	31.9 (19.8-51.2)
2016	23	633	36.4 (24.2-54.7)
2017	21	620	33.8 (22.1-51.9)
Liver transplants			
2011	14	125	111.9 (66.3-188.9)
2012	9	139	64.8 (33.7-124.6)
2013	10	105	95.4 (51.3-177.3)
2014	8	154	51.9 (25.9-103.7)
2015	11	130	84.7 (46.9-153.0)
2016	10	184	54.2 (29.2-100.8)
2017	11	188	58.6 (32.5-105.9)
Thoracic SOT			
2011	9	92	97.4 (50.7-187.3)
2012	7	83	84.0 (40.0-176.1)
2013	6	121	49.6 (22.3-110.4)
2014	11	108	102.0 (56.5-184.2)
2015	6	132	45.6 (20.5-101.5)
2016	13	139	93.6 (54.4-161.3)
2017	14	171	81.9 (48.5-138.2)

© 2021 Hosseini-Moghaddam SM, et al. *JAMA Network Open.*

eTable 6. Baseline contributing factors to posttransplant CDI

Characteristic	Category	Total	CDI (%)	person -time (1000 person -year)	Incidence rate (95% CI)	Hazard ratio (95% CI)	p-value
Age .							
	18 - 49	3904	230 (5.9)	25.7	8.95 (7.86-10.18)	-	-
	50 - 65	5250	364 (6.9)	29.4	12.40 (11.19-13.74)	1.313 (1.113-1.550)	0.0013
	>65	1570	132 (8.4)	6.9	19.06 (16.07-22.61)	1.837 (1.480-2.281)	<.0001
Sex .							
	Male	6901	422 (6.1)	40.1	10.52 (9.56-11.57)	-	-
	Female	3823	304 (8.0)	21.9	13.91 (12.43-15.57)	1.313(1.133-1.522)	0.0003
Donor status §							
	Living	2940	157 (5.3)	20.1	7.81 (6.68-9.13)	-	-
	Deceased	7014	511 (7.3)	38.4	13.32 (12.21-14.53)	1.584 (1.326-1.891)	<.0001
SOT type							
	Kidney	6453	386 (6.0)	40.2	9.60 (8.69-10.60)	-	-
	Heart	523	43 (8.2)	2.8	15.61 (11.58-21.05)	1.546 (1.128-2.118)	0.0067
	Kidney-pancreas	452	33 (7.3)	2.7	12.15 (8.64-17.09)	1.254 (0.879-1.789)	0.2125
	Liver	2117	162 (7.7)	11.6	13.99 (11.99-16.32)	1.400 (1.165-1.682)	0.0003
	Lung	1122	93 (8.3)	4.5	20.59 (16.80-25.23)	1.821 (1.452-2.283)	<.0001
	Multiorgan	57	9 (15.8)	0.2	45.33 (23.58-87.11)	3.860 (1.958-7.611)	<.0001
Income quintile							
	1	2343	165 (7.0)	13.5	12.24 (10.51-14.26)	-	-
	2	2137	154 (7.2)	12.5	12.37 (10.56-14.48)	1.016 (0.816-1.266)	0.8839
	3	2153	144 (6.7)	12.4	11.62 (9.87-13.68)	0.952 (0.761-1.190)	0.6649
	4	2124	140 (6.6)	12.3	11.35 (9.62-13.39)	0.932 (0.744-1.168)	0.5419
	5	1967	123 (6.3)	11.3	10.86 (9.10-12.95)	0.887 (0.703-1.120)	0.3134
Rural status							
	Rural	1146	79 (6.9)	6.8	11.66 (9.35-14.54)	-	-
	Urban	9578	647 (6.8)	55.2	11.72 (10.85-12.66)	0.996 (0.789-1.257)	0.9730
Diabetes							
	Without	6419	368 (5.7)	39.4	9.34 (8.34-10.34)		-
	With	4305	358 (8.3)	22.6	15.85 (14.29-17.59)	1.605 (1.387-1.858)	<.0001
Deyo-Charlson comorbidity							
	2	5215	283 (5.4)	32.1	8.80 (7.83-9.89)	-	-
	3	1589	105 (6.6)	9.7	10.83 (8.94-13.11)	1.227 (0.981-1.534)	0.0734
	>3	3920	338 (8.6)	20.1	16.78 (15.09-18.67)	1.790 (1.527-2.097)	<.0001

© 2021 Hosseini-Moghaddam SM, et al. *JAMA Network Open.*

Pre-transplant CKD							
	Without	3091	216 (7.0)	15.9	13.59 (11.89-15.53)	-	-
	With	7633	510 (6.7)	46.1	11.06 (10.14-12.07)	0.862 (0.735-1.010)	0.0667
Delayed graft function							
	Without	4768	251 (5.3)	30.2	8.30 (7.33-9.39)	-	-
	With	1685	135 (8.0)	10.0	13.54 (11.44-16.03)	1.611 (1.307-1.986)	<.0001
Cancer							
	Without	6842	426 (6.2)	41.7	10.21 (9.29-11.23)	-	-
	With	3882	300 (7.7)	20.3	14.81 (13.22-16.58)	1.370 (1.182-1.589)	<.0001
Inflammatory bowel disease							
	Without	1051<br>1	702 (6.7)	60.7	11.56 (10.74-12.45)	-	-
	With	213	24 (11.3)	1.3	19.07 (12.78-28.46)	1.670 (1.109-2.515)	0.0141
COPD							
	Without	8721	565 (6.5)	52.8	10.70 (9.85-11.61)	-	-
	With	2003	161 (8.0)	9.2	17.58 (15.06-20.51)	1.478 (1.240-1.761)	<.0001
Hospitalization in past 1yr							
	Without	2798	146 (5.2)	18.2	8.02 (6.82-9.44)	-	-
	With	7926	580 (7.3)	43.8	13.25 (12.21-14.37)	1.562 (1.305-1.870)	<.0001
	0	2798	146 (5.2)	18.2	8.02 (6.82-9.44)	.	.
	1-2	4684	288 (6.1)	27.0	10.68 (9.52-11.99)	1.279 (1.049-1.559)	0.0148
	3+	3242	292 (9.0)	16.8	17.35 (15.47-19.46)	2.006 (1.647-2.443)	<.0001
Major abdominal surgeries							
	Without	1007<br>4	694 (6.9)	59.1	11.75 (10.91-12.66)	-	-
	With	650	32 (4.9)	2.9	10.96 (7.75-15.50)	0.833 (0.584-1.188)	0.3139
Pre-transplant CDI							
	Without	1063<br>1	711 (6.7)	61.6	11.55 (10.73-12.43)	-	-
	With	93	15 (16.1)	0.4	37.05 (22.34-61.46)	2.928 (1.711-5.011)	<.0001
NG tube in past 1 year							
	Without	1069<br>4	723 (6.8)	61.8	11.69 (10.87-12.58)	-	-

	With	37	≤6	-	18.13 (5.85-56.21)	1.417 (0.455-4.414)	0.5476
Mechanical ventilation in past 1 year							
	Without	1030 2	680 (6.6)	60.2	11.30 (10.48-12.18)	-	-
	With	422	46 (10.9)	1.8	25.57 (19.15-34.14)	2.026 (1.501-2.737)	<.0001
Days of hospital stay after index date							
	<21	8828	457 (5.2)	53.9	8.48 (7.74-9.29)	-	-
	21 - 42	1225	148 (12.1)	6.0	24.50 (20.85-28.78)	2.698 (2.239-3.251)	<.0001
	>42	671	121 (18.0)	2.1	58.82 (49.22-70.30)	5.484 (4.473-6.724)	<.0001
Length of index episode							
	<21	8377	422 (5.0)	51.5	8.19 (7.44-9.01)	-	-
	21 - 42	1324	144 (10.9)	6.6	21.82 (18.53-25.69)	2.484 (2.055-3.003)	<.0001
	>42	1023	160 (15.6)	3.9	41.43 (35.49-48.38)	4.322 (3.599-5.190)	<.0001

§ Missing information

Donor status: n=770

Income quantile: n=32

© 2021 Hosseini-Moghaddam SM, et al. *JAMA Network Open.*

eReference

1. CST. Canadian Society of Transplantation - Programs & OPO’s. https://www.cst-transplant.ca/cgi/page.cgi/transplant-programs-opos.html. Published 2021. Accessed April 7, 2020.

© 2021 Hosseini-Moghaddam SM, et al. *JAMA Network Open.*

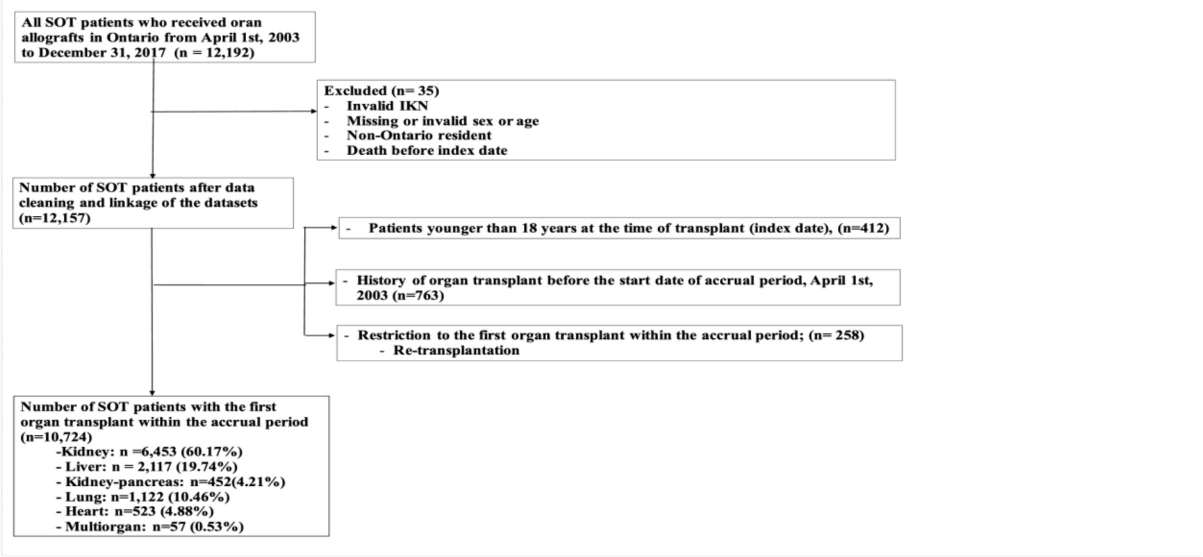

eFigure 1- Study flowchart

© 2021 Hosseini-Moghaddam SM, et al. *JAMA Network Open.*

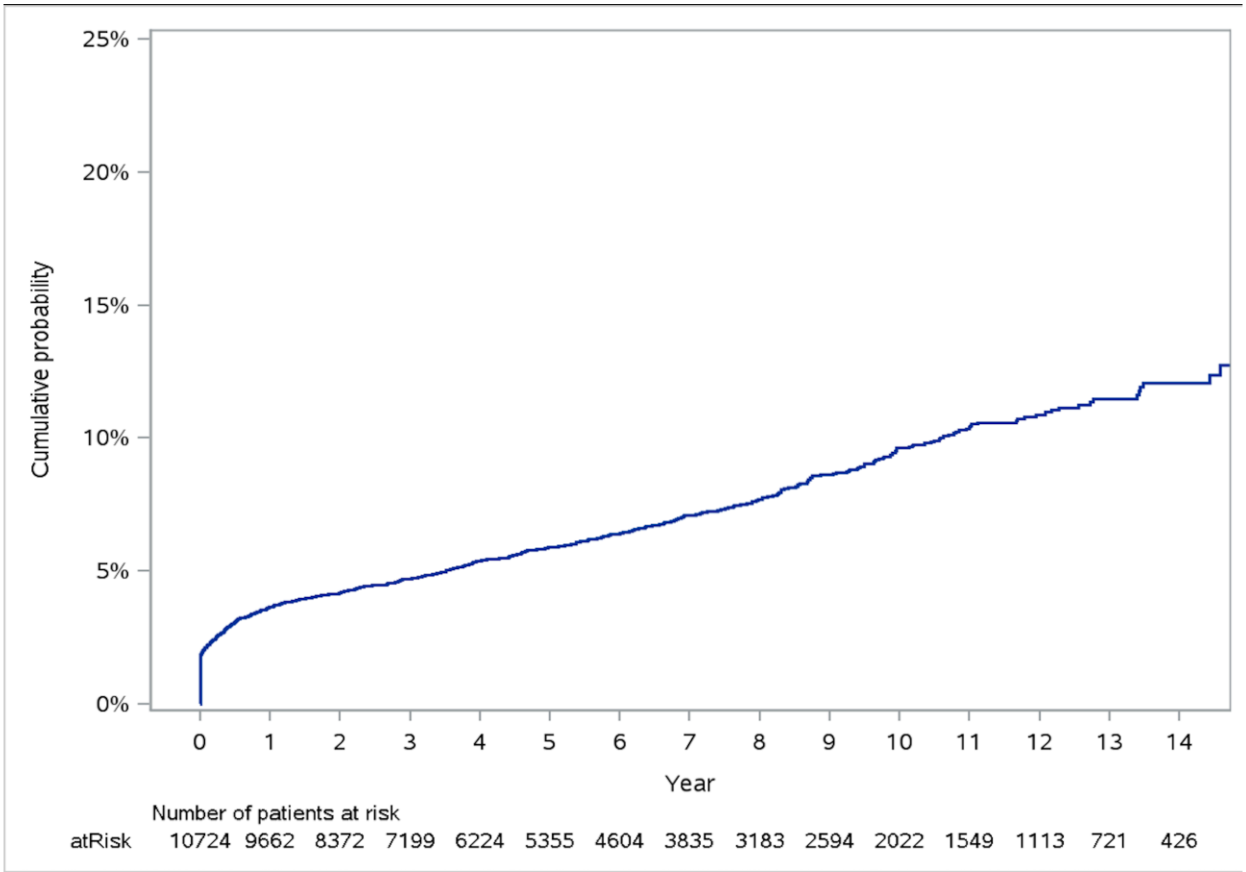

eFigure 2- Cumulative probability of CDI for all SOT recipients

© 2021 Hosseini-Moghaddam SM, et al. *JAMA Network Open.*
